# IL-1β promotes stemness and invasiveness of colon cancer cells through Zeb1 activation

**DOI:** 10.1186/1476-4598-11-87

**Published:** 2012-11-23

**Authors:** Yijing Li, Lei Wang, Loretta Pappan, Amy Galliher-Beckley, Jishu Shi

**Affiliations:** 1 Department of Anatomy and Physiology, College of Veterinary Medicine, Manhattan, KS 66506, USA

**Keywords:** Colon cancer, Tumor microenvironment, Inflammation, Interleukin-1β, Cancer stem cells, Epithelial-mesenchymal transition, Zeb1

## Abstract

**Background:**

IL-1β is a pleiotropic pro-inflammatory cytokine and its up-regulation is closely associated with various cancers including gastrointestinal tumors. However, it remains unclear how IL-1β may contribute to the initiation and development of these inflammation-associated cancers. Here we investigated the role of IL-1β in colon cancer stem cell (CSC) development.

**Methods:**

Using self-renewal assay, soft-agar assay, invasion assay, real-time PCR analysis, immunoblot assay and shRNA knockdown, we determined the effects of IL-1β on cancer stem cell development and epithelial-mesenchymal transition (EMT) in human primary colon cancer cells and colon cancer cell line HCT-116.

**Results:**

We found that IL-1β can increase sphere-forming capability of colon cancer cells in serum-free medium. IL-1β-induced spheres displayed an up-regulation of stemness factor genes (Bmi1 and Nestin) and increased drug resistance, hallmarks of CSCs. Importantly, expression of EMT activator Zeb1 was increased in IL-1β-induced spheres, indicating that there might be a close association between EMT and IL-1β-induced CSC self-renewal. Indeed, IL-1β treatment led to EMT of colon cancer cells with loss of E-cadherin, up-regulation of Zeb1, and gain of the mesenchymal phenotype. Furthermore, shRNA-mediated knockdown of Zeb1 in HCT-116 cells reversed IL-1β-induced EMT and stem cell formation.

**Conclusion:**

Our findings indicate that IL-1β may promote colon tumor growth and invasion through activation of CSC self-renewal and EMT, and Zeb1 plays a critical role in these two processes. Thus, IL-1β and Zeb1 might be new therapeutic targets against colon cancer stem cells.

## Background

Chronic inflammation is a predisposing cause of various cancers. Interestingly, inflammatory cells and mediators are present in every tumor, including those that are not developed from chronic inflammation [1,2]. The inflammatory microenvironment around the tumor is a critical component that drives tumor progression, and it is often characterized as the seventh hallmark of cancer [3]. Interleukin-1β (IL-1β) is an important mediator of cancer-related inflammation and can be secreted by immune, stromal and tumor cells [4]. IL-1β levels are increased in a variety of cancers including colon cancer, one of the most common fatal cancers [5,6]. Recent studies have shown that the interaction between colon cancer cells and immune cells induces secretion of IL-1β from immune cells [7,8]. Elevated IL-1β levels have been associated with increased colon tumor growth and invasion [9,10]. However, how IL-1β may contribute to the development of cancer has not been fully explored.

Cancer stem cells (CSCs) are a subpopulation of tumor cells with the ability to undergo self-renewal and recapitulate the entire tumor population *in vitro* and *in vivo*[11]. Similar to CSCs from other types of cancers, colon CSCs have been identified from human colon tumor cells using flow cytometry and measuring the expression of stem cell markers [12-14]. The ability to form spheroid cultures in serum-free conditions supplemented with growth factors is also used for identification and expansion of CSCs *in vitro*[12,15]. In addition to the capability of self-renewal, CSCs have the ability to initiate distant metastasis to form metastatic growth that resemble the primary tumors, and are resistant to conventional chemotherapy/radiotherapy, implicating that they are responsible for tumor growth and recurrence [16]. Recently, we and others have shown that soluble factors within tumor microenvironment play an important regulatory role in the self-renewal and fate of CSCs [17,18]. We speculate that IL-1β may promote tumor growth by increasing the self-renewal capability of colon CSCs.

Epithelial-mesenchymal transition (EMT) is a process which involves epithelial cells acquiring a mesenchymal phenotype and migratory capability, and plays an important role in tumor metastasis [19,20]. EMT can be triggered by various extracellular stimuli and microenvironment factors. The induction of EMT is mediated by a set of key transcription factors within the cell, including Twist, Snail, Snug, Zeb1 and Zeb2, many of which are frequently over-expressed in cancer cells [20]. These EMT activators directly repress the expression of E-cadherin, an integral component of adherens junctions. Importantly, EMT process has been associated with the acquisition of stem cell properties in normal and cancer cells [21,22]. The link between EMT and CSCs enables cancer cells to migrate from the primary tumor and colonize distant sites.

In this study, we investigated whether IL-1β could promote stem cell and EMT phenotypes in human colon HCT-116 cells as well as in newly established primary colon cancer cells. HCT-116 cells are a well-characterized cellular model of human colon cancer and have been broadly used for colon cancer research. The low passages of freshly isolated human colon cancer cells have allowed us to closely mimic the *in vivo* state and generate more physiologically relevant data. Here, we provide direct evidence that IL-1β promotes self-renewal of colon cancer cells as well as their acquisition of EMT phenotype, and this induction of CSC and EMT phenotypes was mediated by Zeb1.

## Results

### Characterization of human primary colon cancer cells

During the first week of culture, all cells freshly isolated from normal mucosa failed to survive, and the majority of cells isolated from colon tumor sections died as well, leaving only a few remaining healthy cells. The initial primary colon cancer cells grew slowly and the culture was maintained for four weeks before they were split for the first passage. Early passages (passage 1–3) of human primary colon cancer (HPCC) cells were used in all experiments in this study.

**Figure 1:**
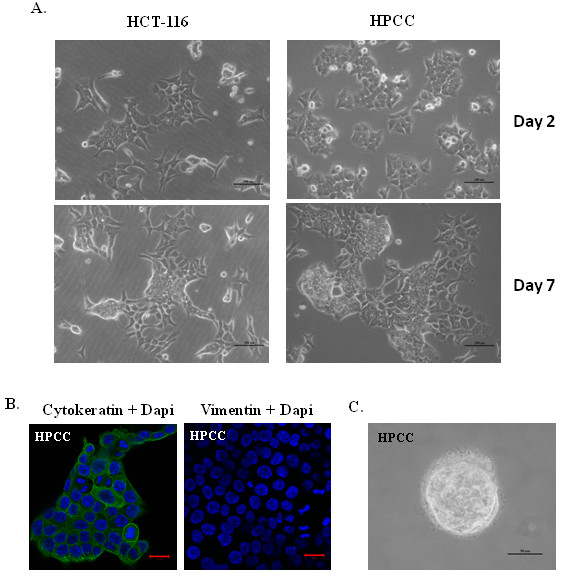
**Characterization of human primary colon cancer cells (HPCC). **(**A**) HPCC cells exhibit epithelial-like morphology as human colon cancer cell line HCT-116. During initial growth in culture (Upper panel), HPCC cells formed small islets; while in late stage of growth (lower panel) they formed multilayer culture, similar to HCT-116 cells. Scale bar = 200 μm. (**B**) HPCC cells react only with cytokeratin but not vimentin as determined using an immunofluoresent staining assay. Nuclei (blue staining) were counterstained with DAPI. Scale bar = 20 μm. (**C**) HPCC display transformation capability as determined using a soft-agar assay. HPCC cells (1x10^4^/well) were plated in soft agar containing the medium with 10% FBS in 6-well plates for 14 days. Scale bar = 50 μm.

The morphology of HPCC cells was first examined using a light phase-contrast microscope. During the first few days after plating, HPCC cells proliferated and formed polarized epithelial islets. While in advanced growth (after seven days), HPCC cells grew as multilayers and formed tumor-like cell clusters, similar to the morphology of colon cancer cell line HCT-116 (Figure 1A). The HPCC culture contained epithelial cells and did not have fibroblasts as determined by immunofluorescence staining with anti-pan-cytokeratin (an epithelial cell marker) and anti-vimentin (a fibroblast cell marker), in which cells reacted only with anti-pan-cytokeratin and were negative for anti-vimentin (Figure 1B).

To assess tumorigenic potential of HPCC cells, we performed a colony-forming assay that is frequently utilized for *in vitro* evaluation of malignant transformation and tumorigenesis [23]. HPCC cells exhibited transforming capability by forming colonies over the period of two weeks (Figure 1C).

### IL-1β promotes sphere formation, expression of stemness markers, and drug resistance

**Figure 2:**
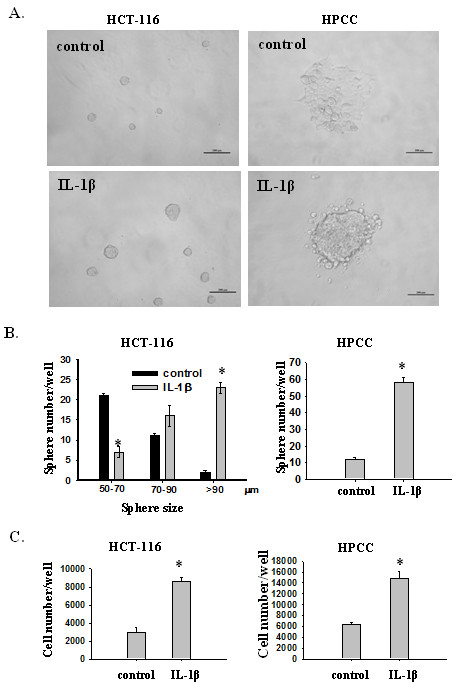
**IL-1β induces sphere formation and proliferation of colon cancer cells in serum-free medium (SFM). **(**A**) Representative images of HCT-116 and HPCC cells in the absence or presence of IL-1β. HCT-116 and HPCC cells (1 cell/μl) were cultured in 96-well plates containing 100 μl SFM in each well with or without IL-1β for seven days. The number of spheres was counted under a microscope and size was measured using ImageJ (**B** and **C**). Then sphere cells were dissociated and stained with Trypan blue and counted under a microscope. Error bars represent SEM. *p < 0.05.

To investigate whether IL-1β promotes self-renewal of colon cancer cells, an important feature of CSCs, we plated HCT-116 and HPCC cells at very low densities in 96-well plates containing serum-free medium with or without IL-1β. The newly formed spheres were then measured and total number of cells was counted. HCT-116 cells grew readily as spheres in serum-free medium. The presence of IL-1β markedly increased the number and size of spheres (Figure 2A &2B, left panel), as well as the total number of cells (Figure 2C, left panel), compared to that of control HCT-116 cells. Unlike HCT-16 cells, most HPCC cells grew as a monolayer in serum-free medium. However, addition of IL-1β to the medium resulted in increased sphere formation (Figure 2A &2B, right panel) and cell proliferation (Figure 2C, right panel). As sphere formation and growth represent the ability of cell self-renewal, these data suggest that IL-1β promotes self-renewal capacity in colon cancer cells, a critical characteristic of CSCs.

**Figure 3:**
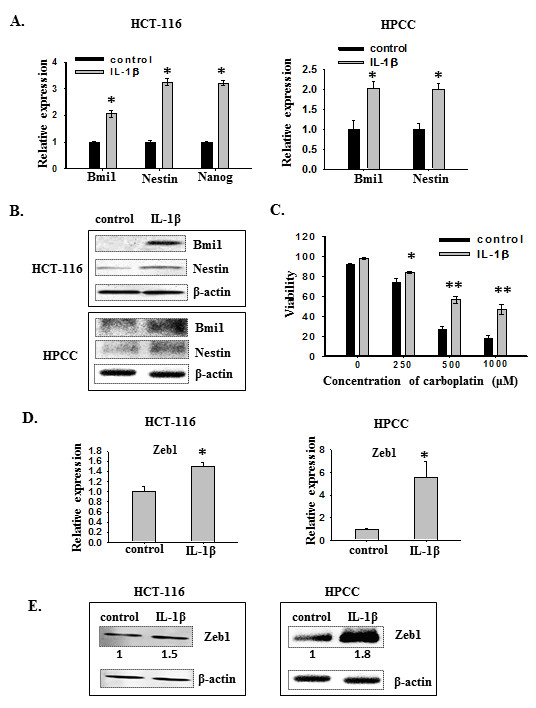
**IL-1β-induced sphere cells express stem cell markers and Zeb1. **(**A**) Transcription levels of stem cell markers in control and IL-1β-treated colon cancer cells. HCT-116 and HPCC cells were cultured in SFM with or without IL-1β for seven days. mRNA levels of stem cell markers were determined by real-time PCR. β-actin was used as an internal normalization control. Error bars represent SEM. *p < 0.05. (**B**) Immunoblot analysis for Bmi1 and Nestin from control and IL-1β-treated cells. β-actin was used as an sample loading control. (**C**) IL-1β-induced sphere cells develop drug resistance. The control and IL-1β-induced sphere cells were treated with various concentrations of carboplatin for two days. Then, cells were dissociated and stained with Trypan blue, and counted under a microscope. The viability was determined by the percentage of live cells over the sum of live and dead cells. *p < 0.05, **p < 0.01. (**D**). Transcription levels of Zeb1 in control and IL-1β-treated colon cancer cells. HCT-116 and HPCC cells were cultured in SFM with or without IL-1β for seven days. mRNA levels of Zeb1 were determined by real-time PCR. β-actin was used as an internal normalization control. Error bars represent SEM. *p < 0.05. (**E**). Immunoblot analysis for Zeb1 from control and IL-1β-treated cells. β-actin was used as an sample loading control. Normalized quantification of band intensities on immunoblots for Zeb1 is listed under each band.

To further characterize IL-1β-induced spheres and determine the molecular mechanisms underlying these observations, we evaluated the expression of several stemness genes in HCT-116 and HPCC cells treated with or without IL-1β in serum-free medium using real time PCR (Figure 3A). We found that the expression of Bmi1, nestin and Nanog were significantly up-regulated in IL-1β-treated HCT-116 cells, compared to that in control cells. Likewise, the expression of Bmi1 and Nestin was increased in IL-1β-induced HPCC sphere cells as well (Figure 3A). The enhanced expression of Bmi1 and nestin in IL-1β-treated HCT-116 cells and HPCC cells was further confirmed by immunoblotting (Figure 3B). These results indicate that IL-1β-induced spheres contain enriched population of CSCs.

Recent studies suggest that CSCs exhibit intrinsic resistance to conventional chemotherapies [24]. Thus, we evaluated whether IL-1β-promoted HCT-116 sphere cells could display enhanced drug resistance against carboplatin, a conventional chemotherapeutic agent. HCT-116 cells were cultured in serum-free medium with or without IL-1β for five days and then exposed to various concentrations of carboplatin for two days. Cell viability was measured as the percentage of live cells relative to total cell number (dead + live cells). As shown in Figure 3C, IL-1β-treated sphere cells displayed significantly greater resistance to carboplatin at concentrations range from 250 to 1000 μM.

To assess whether IL-1β-induced formation of CSCs was associated with EMT, we examined the expression of EMT activators using real-time PCR and western blot analyses. As shown in Figure 3D and 3E, Zeb1 expression (gene transcript and protein) were augmented in IL-1β-induced HCT-116 and HPCC sphere cells in serum free medium. Interestingly, although the magnitude of induction of Zeb1 mRNA by IL-1β in HPCC cells was markedly greater than that in HCT-116 cells (5.5 fold vs. 1.5 fold, Figure 3D), the difference in Zeb1 protein induction by IL-1β was not that dramatic between HPCC and HCT-116 cells (1.8 fold vs. 1.5 fold, Figure 3E). Nonetheless, this result suggests that Zeb1 may be involved in IL-1β-mediated reactivation or de novo induction of cancer stem cells.

### IL-1β induces an EMT phenotype and enhances invasiveness of colon cancer cells

**Figure 4:**
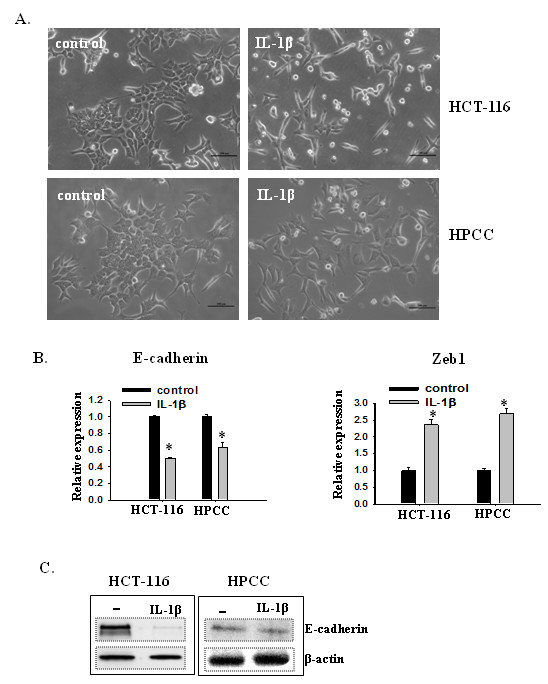
**IL-1β induces EMT in colon cancer cells. **(**A**) IL-1β induces morphological changes from epithelial-like to fibroblast-like appearances in colon cancer cells. HCT-116 and HPCC cells were cultured in the medium with 1% FBS in the presence or absence of IL-β for seven days. Scale bar = 200 μm. (**B**) Transcription levels of Zeb1 and E-cadherin as determined by real-time PCR. β-actin was used as an internal normalization control. Error bars represent SEM. *p < 0.05. (**C**) Immunoblot analysis for E-cadherin from control and IL-1β-treated cells. β-actin was used as an sample loading control.

Since Zeb1, a transcription factor that mediates EMT, was up-regulated in IL-1β-induced stem cells in serum free medium, we then investigated whether IL-1β could induce EMT phenotype in colon cancer cells. HCT-116 and HPCC cells were cultured in McCoy’s 5A Medium with 1% FBS in the presence or absence of 200 pM IL-1β for seven days. In the absence of IL-1β, HCT-116 and HPCC cells displayed an epithelial cell phenotype and formed islets. In contrast, in the presence of IL-1β both groups of cells acquired a more fibroblast-like, spindle-shaped morphology indicative of mesenchymal cells (Figure 4A). The morphological transformation was consistent with reduction of E-cadherin expression in IL-1β-treated cells, as analyzed by rt-PCR and immunoblotting (Figure 4B &4C). To identify the factors that mediated IL-1β-induced EMT in colon cancer cells, we examined the expression patterns of E-cadherin transcriptional repressors using rt-PCR. It was found that the expression of Zeb1 (Figure 4B), but not Snail or Twist (data not shown), was up-regulated in HCT-116 cells and HPCC EMT cells.

**Figure 5:**
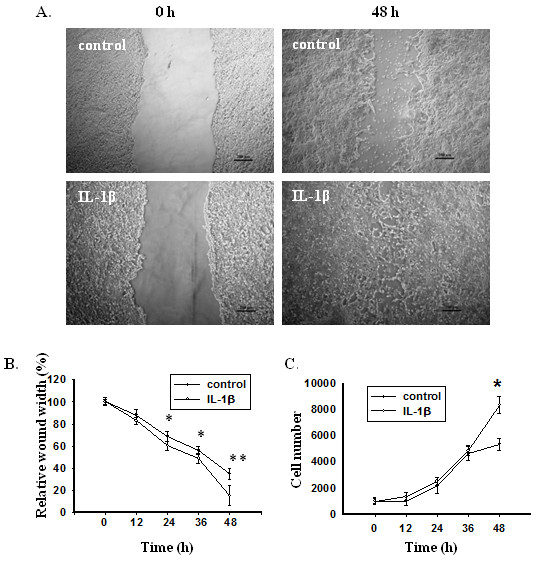
**IL-1β enhances invasiveness and proliferation of colon cancer cells. **(**A**) Representative images of wounds at 0 and 48 h in the presence or absence of IL-1β. Confluent monolayers of HCT-116 cells were scraped by a pipette tip to generate wounds and then were cultured in the presence or absence of IL-1β for 48 h. (**B**) Relative wound width represented as percentages compared to the wound width at 0 h. (**C**) Proliferation of HCT-116 cells in the presence or absence of IL-1β as measured by cell count. Error bars represent SEM. *p < 0.05.

To determine whether IL-1β-induced EMT cells had increased invasiveness as conferred by EMT, a wound healing assay was used to assess cell migration. An area devoid of cells was created at time 0 by scraping a monolayer of HCT-116 cells and followed by incubation of the cells with or without IL-β for 48 h. Compared to control cells, IL-β-treated HCT-116 cells showed a significantly (p < 0.05) higher rate of migration starting from 24 h and the leading edges along the scraped area had almost coalesced 48 h after scraping (Figure 5A &5B).

The proliferation status of HCT-116 cells grown under the same conditions was also assessed at the indicated time after plating (Figure 5C). As shown in Figure 5C, IL-1β-treated cells proliferated at the same rates as control cells between 0–36 h time point, but significantly (p < 0.05) more rapidly than control cells after 36 h. This result indicates that enhanced proliferation may have contributed to the faster wound closure of IL-1β-treated culture at late stage, but not during the early-to-mid stage of wound healing.

### IL-1β-induced EMT in HCT-116 cells is mediated by Zeb1

**Figure 6:**
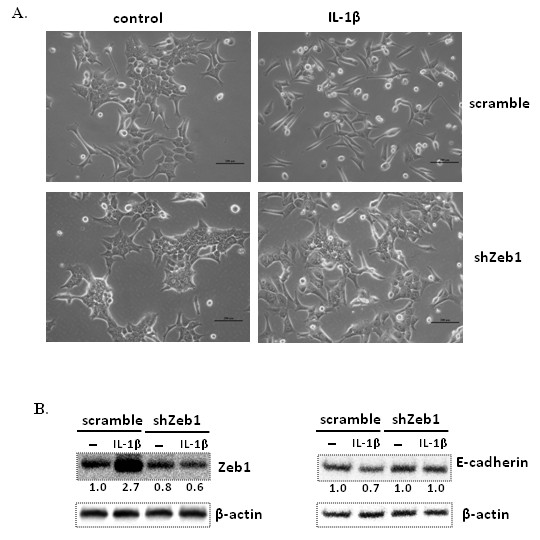
**Knockdown of Zeb1 inhibits IL-1β-induced EMT in HCT-116 cells. **(**A**) Zeb1 knockdown cells still maintain epithelial appearance seven days after treatment with IL-1β. Scale bar = 200 μm. Scramble and shZeb1 HCT-116 cells were cultured in the medium with 1% FBS in the presence or absence of IL-1β for seven days. (**B**) Zeb1 knockdown inhibits IL-β-induced suppression of E-cadherin expression. Immunoblot analysis for Zeb1 and E-cadherin was performed on lysates from control and IL-1β-treated cells. β-actin was used as an sample loading control. Normalized quantification of band intensities on immunoblot for Zeb1 and E-cadherin is listed under each band.

To determine whether IL-1β-induced self-renewal and EMT in HCT-116 cells were due to the up-regulation of Zeb1, we sought to selectively suppress the expression of Zeb1 using shRNA. HCT-116 cells were transduced with lentiviral vectors carrying scrambled shRNA or specific shRNAs against Zeb1. We first examined the impact of Zeb1 knockdown on IL-1β-induced EMT. The two stable shRNA pools expressing scrambled and Zeb1 shRNAs (shZeb1) were cultured in MCCOY’S 5A MEDIUM with 1% FBS in the presence or absence of IL-1β for seven days. As shown in Figure 6A, HCT-116 cells treated with shZeb1 demonstrated a more epithelial phenotype in the presence of IL-1β, which was similar to that displayed by HCT-116 cells in the absence of IL-1β regardless whether they were treated with shZeb1 or the scramble shRNA. In contrast, HCT-116 cells treated with scramble shRNA displayed spindle-shape morphology after IL-1β stimulation, similar to that of control (without shRNA treatment) HCT-116 cells after IL-1β stimulation (Figure 4A). This observation is consistent with the notion that Zeb1 might be responsible for IL-1β-induced EMT in HCT-116 cells.

To further verify the interaction of Zeb1 and E-cadherin in IL-1β-induced EMT in HCT-116 cells, we first measured the expression of Zeb1 in HCT-116 cells treated with scrambled shRNA or shZeb1. As shown in Figure 6B, Zeb1 protein expression in shZeb1-treated cells was reduced compared to that in HCT-116 cells treated with scramble shRNAs in the absence of IL-1β, and its expression was not increased in the presence of IL-1β. In contrast, Zeb1 expression in HCT-116 cells treated with scrambled shRNA was increased 2.7 fold after IL-1β treatment (Figure 6B, left panel). This clearly indicates that the IL-1β-induced Zeb1 was knocked down effectively. Because the expression of E-cadherin in shZeb1-treated cells did not change in the presence or absence of IL-1β (Figure 6B), our data support the notion that Zeb1 is indeed responsible for IL-β-induced suppression of E-cadherin in HCT-116 cells.

### IL-1β-induced CSC self-renewal in HCT-116 cells is mediated by Zeb1 and Bmi-1

**Figure 7:**
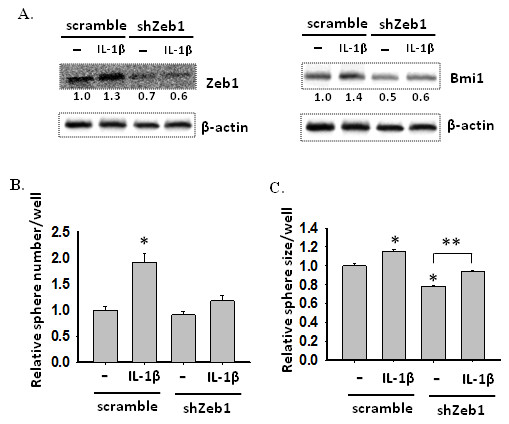
**Zeb1 knockdown decreases Bmi1 expression as well as sphere-forming capability in HCT-116 cells treated with or without IL-1β. **(**A**) Zeb1 knockdown inhibits Bmi1 expression in either IL-1β-treated or untreated cells. Scramble and shZeb1 HCT-116 cells were cultured in SFM in the presence or absence of IL-1β for seven days. Expression of Zeb1 and Bmi1 was determined using immunoblot assay on lysates from scramble and shZeb1 cells treated with or without IL-1β. β-actin was used as an sample loading control. Normalized quantification of band intensities on immunoblot for Zeb1 and Bmi1 is listed under each band. (**B**) Zeb1 knockdown reduces sphere-forming capability in HCT-116 cells as well as inhibits IL-1β-induced sphere formation. Scramble or shZeb1 cells (1 cell/μl) were cultured in 96-well plates containing 100 μl SFM in each well with or without IL-1β for seven days. The number of spheres was counted under a microscope and size was measured using ImageJ. The data are presented as relative sphere number and size as compared to scramble cells cultured in the absence of IL-1β. Error bars represent SEM. *p < 0.05.

To determine whether Bmi-1 is involved in IL-1β-Zeb1-mediated CSC development, we evaluated the effect of Zeb1 knockdown on IL-1β-induced Bmi1 expression and self-renewal of HCT-116 cells. Cells were cultured in serum-free medium in the presence or absence of IL-1β for seven days and the expression of Zeb1 and Bmi1 were examined by immunoblotting. As expected, Zeb1 protein expression was enhanced in scramble cells after IL-1β treatment, but was reduced in shZeb1 cells with or without IL-1β, compared to scramble cells without IL-1β treatment (Figure 7A). Similarly, Bmi1 expression in shZeb1 cells was remarkably decreased compared to scramble cells, and still remained at the low level after IL-1β treatment. The parallel expression patterns between Zeb1 and Bmi1 indicates that Zeb1 may regulate the expression of Bmi1 in HCT-116 cells treated with IL-1β.

To determine the effect of Zeb1 knockdown on self-renewal of HCT-116 cells, scramble and shZeb1 cells were cultured at a very low density (1 cell/μl) in 96-well plates containing serum-free medium with or without IL-1β for seven days. In the absence of IL-1β, Zeb1 knockdown cells showed reduced self-renewal capability by forming smaller sized but similar numbers of spheres compared to scramble cells (Figure 7B &7C). The reduced self-renewal capability of Zeb1 knockdown cells is comparable to their reduced Bmi-1 expression (Figure 7A). IL-1β treatment led to a significant increase in the number and size of spheres formed from scramble cells, paralleled with their increased Bmi1 expression. As for Zeb1 knockdown cells, IL-1β failed to significantly increase the number of spheres, but the size of these spheres was significantly (p < 0.05) increased (Figure 7B &7C). The limited effect of Zeb1 knockdown on IL-1β-mediated sphere size increase suggests that in addition to Zeb1, other factors may also contribute to IL-1β-enhanced self-renewal capability. However, the significant inhibition of Zeb1 knockdown on IL-1β-mediated increase of sphere number suggests that Zeb1-Bmi1 pathway is indeed involved in regulation of IL-1β-induced self-renewal.

## Discussion

IL-1β is a pleiotropic cytokine with numerous roles in various physiological and pathological states. Aberrant production and signaling of IL-1β are tightly linked to tumor generation, growth and metastasis in multiple types of cancers [25-29]. Thus far, the exact mechanisms by which IL-1β promotes tumor growth have remained unclear. Using a colon cancer cell line HCT-116 and primary colon cancer cells, we have found that IL-1β can promote sphere-forming capacity concomitant with up-regulated expression of stemness markers Bmi1 and nestin in colon cancer cells, suggesting that IL-1β increases the self-renewal of colon CSCs. In addition, IL-1β-induced spheres display augmented drug resistance, a property associated with CSCs. Importantly, IL-1β induces cellular morphological changes in colon cancer cells that are consistent with the acquisition of EMT phenotype as characterized by the loss of E-cadherin expression. Furthermore, IL-1β-induced EMT cells display enhanced migratory capacity compared with parental cells with an epithelial phenotype. Overall, our studies provide the first evidence that IL-β promotes CSC self-renewal and EMT in colon cancer cells, which may contribute to colon cancer growth, metastasis and recurrence.

Bmi-1 is a transcriptional repressor belonging to the polycomb group protein family, which functions in gene silencing through chromatin modification [30,31]. Bmi-1 plays a crucial role in the self-renewal of normal and neoplastic stem cells [32-37]. In addition to Bmi-1, several other molecules have been proposed as colon CSC markers including CD133, CD44, Lgr-5 and pluripotency genes such as Oct-4, Sox-2 and Nanog [38]. However, recent studies by others have shown that NANK, a human colon tumor cell line with a high level of Bmi-1 expression but negative for CD133, CD44, Oct4, and Nanog, is capable of initiating tumors in mice [39]. This report suggests that Bmi-1 is crucial for the self-renewal and oncogenic potential of colon CSCs. In addition, aberrant expression of Bmi-1 has been reported in human colon cancer and its expression levels correlate with clinical and pathological stages of colon malignancies, further assuring the oncogenic role of Bmi-1 in colon cancer [40-42]. Here we have found that the enhanced sphere formation and proliferation in HCT-116 and HPCC cells treated with IL-1β are associated with significantly augmented Bmi-1 expression in these cells. This observation supports the notion that IL-1β acts through Bmi-1 to promote the self-renewal and proliferation of colon cancer stem cells.

Zeb1, Zeb2 and transcription factors such as Snail, Slug, E47 and Twist are all able to activate EMT through binding to the E-cadherin promoter and repressing its transcription [43]. In this study, we found that the expression of Zeb1, but not Zeb2, Snail or Twist, was up-regulated in IL-1β-induced HCT-116 and HPCC EMT cells. This indicates that IL-1β may act through Zeb1 to induce EMT in colon cancer cells. The importance of Zeb1 in IL-1β-induced EMT in colon cancer cells is further highlighted in Zeb1 knockdown HCT-116 cells, which display a close association between the lack of EMT and the disappearance of IL-1β-induced repression of E-cadherin transcription in these cells (Figure 6). Interestingly, IL-1β-induced Zeb1 expression has also been observed in head and neck squamous carcinoma cells [44]. Thus, Zeb1 may play a key role in IL-1β-induced EMT in various cancer cells.

It has been reported that Zeb1 links the EMT process and acquisition of CSC properties through a double-negative feedback loop with microRNA-200, a repressor of Bmi1 expression [45-48]. Our results show that Zeb1 was also up-regulated along with Bmi1 in IL-1β-induced HCT-116 and HPCC spheres. Thus, the parallel up-regulation of Zeb1 and Bmi1 by IL-1β suggests that IL-β-induced self-renewal of colon cancer cells may involve the Zeb1-Bmi1 pathway. This notion is supported by the behavior of Zeb1 knockdown HCT-116 cells, in which Bmi1 expression is reduced in the absence of IL-1β and its expression is not increased in the presence of IL-1β, compared to that in control cells (Figure 7A). In addition, Zeb1 knockdown cells displayed reduced self-renewal capacity in the absence or presence of IL-1β, compared to that of control cells (Figure 7B).

Although our data support the hypothesis that Zeb1-Bmi1 pathway is critical for IL-β-induced self-renewal of colon CSCs, we cannot rule out the possibility that other stemness factors are also involved in IL-β-induced self-renewal. This is because Zeb1 knockdown cells still demonstrate obvious increases in the self-renewal capacity after IL-β treatment. Thus, we speculate that IL-1β enhances self-renewal of colon CSCs through activating a multifaceted stemness system in which the Zeb1-Bmi1 pathway is a part of an essential network.

## Conclusion

EMT enables tumor metastasis and CSC self-renewal is a key driving force for tumor growth and recurrence after chemotherapy. IL-1β is a major pro-inflammatory cytokine that has been closely linked to enhanced growth and invasion of colon cancer. Using a colon cancer cell line and primary colon cancer cells, we have demonstrated that IL-1β may act via Zeb1 to promote EMT and stem cell development in colon cancer cells, which may contribute to colon tumor malignancy. Thus, IL-1β and Zeb1 can be potential therapeutic targets aimed at cancer stem cells in the development of novel treatments for colon cancer.

## Methods

### Isolation of primary colon cancer cells and cell culture

Tumor samples and corresponding normal mucosa from a patient with stage 2 colon cancer were obtained from the Surgical Associates, Manhattan, KS. The proposal (#5753) to isolate human colon cancer cells was reviewed and approved by the Institutional Review Board (IRB) for Kansas State University. At operation, collected tissues were placed in McCoy’s 5A Medium with 10% fetal bovine serum (FBS) and 1% penicillin-streptomycin (Invitrogen Corp, Carlsbad, CA) on ice and transported to the laboratory immediately. The fresh tissue was cut into small pieces (1 mm) using scissors and digested with 3 ml (0.4 mg/ml) Collagenase A (Roche Applied Science, Indianapolis, IN) at 37°C for 1 h with interval agitation. Twenty microliter of EDTA (500 mM) was used to stop the reaction. The dispersed tissues were quickly mixed in a homogenizer and the mixture was filtered through a 70 μm strainer (BD Falcon, San Jose, CA) to remove tissue fragments. Cells were washed with PBS, spun down by centrifugation and cultured in McCoy’s 5A Medium with 10% FBS and 1% penicillin-streptomycin in a humidified incubator with 37°C and 5% CO_2_.

Human colon cancer cell line HCT-116 was obtained from the American Type Culture Collection (Manassas, VA) and maintained in McCoy’s 5A Medium with 10% FBS and 1% penicillin-streptomycin. To induce EMT, HCT-116 cells and primary colon cancer cells HPCC were cultured in McCoy’s 5A Medium with 1% FBS in the presence 200 pM IL-1β (R&D Systems, Minneapolis, MN) for seven days. To induce sphere formation, cells were cultured in serum-free medium (SFM) which consisted of neurobasal-A medium supplemented with B27, GlutaMAX-I supplement, 1% penicillin-streptomycin (all from Invitrogen Corp), 50 ng/ml heparin (Sigma-Aldrich, Saint Louis, MO), 20 ng/ml of EGF, and 20 ng/ml bFGF (R&D systems, Minneapolis, MN). To determine the effect of IL-1β, 200 pM IL-1β was added to the serum-free medium every other day.

### Immunostaining

Primary colon cancer cells were seeded in slide chambers (Fisher Scientific, Hanover Park, IL) and cultured for seven days in McCoy’s 5A Medium with 10% FBS. Then, cells were fixed with 4% paraformaldehyde, permeablized with PBS containing 0.5% Triton X-100, and incubated with pan-cytokeratin (C 2562, Sigma) or vimentin (sc-6260, Santa Cruz Biotechnology, Santa Cruz, CA) mouse monoclonal antibodies, and followed by secondary chicken anti-mouse IgG (H + L) antibody conjugated with Alexa 488 (Invitrogen). Cells were then mounted with VECTASHIELD Mounting Medium with DAPI (Vector laboratories, Burlingame, CA) and observed with a confocal microscope.

### Soft agar colony formation assay

Anchorage independent growth in soft agar was used to determine the transformation and growth of the primary colon cancer cells *in vitro*. The soft agar assay was performed in 6-well plates containing two layers of Sea Plague Agar (Invitrogen). The bottom layer consisted of 0.8% agar in 1 ml of McCoy’s 5A Medium with 10% FBS. The primary colon cancer cells (1x10^4^/well) were placed in the top layer containing 0.4% agar in the same medium as the bottom. Cells were cultured for 14 days and colonies were photographed under a microscope.

### Chemoresistance assay

Control monolayer and IL-1β-induced sphere cells in serum-free medium were treated with carboplatin (Sigma-Aldrich) at concentrations of 250, 500, 1000 μM for two days. Then cells were stained with Trypan blue (Amresco Inc., Solon, OH) and counted under a microscope. The viability of the cells was measured as the percentage of live cells over the total of live and dead cells.

### Wound healing assay

HCT-116 cells were cultured for four days in 6-well plates containing McCoy’s 5A Medium plus 10% serum to generate a confluent monolayer. The media was then removed and two wounds per well were made by scraping with pipette tips. The wounds were examined to ensure that the cells were removed completely. The plates were washed twice with PBS to remove cellular debris and then McCoy’s 5A Medium with 1% serum with or without IL-1β was added. Pictures from the same area of the wound were taken under a microscope at 0, 12, 24 and 48 h after scraping. For each wound, the distance of the gap was the average of four fields. The measurement for six wounds per treatment were collected and analyzed statistically.

### Self-renewal assay and cell proliferation assay

HCT-116 cells (1 cell/μl in SFM) and primary colon cancer cells (1 cell/μl in SFM) were seeded at 100 μl/well in 96-well plates and treated with or without 200 pM IL-1β for seven days. IL-1β was added every other day. The total number of spheres in each well was counted under a microscope. Then cells were dissociated, stained with Trypan blue (Amresco Inc., Solon, OH) and counted under a microscope to determine the total cell number.

### RNA extraction and real-time PCR

Total RNA was extracted using TRI reagent (Sigma-Aldrich), followed by digestion with a DNase kit (Applied Biosystems, Carlsbad, California) to remove DNA residues. Reverse transcription was carried out using the iScript cDNA synthesis kit (Bio-Rad, Hercules, CA) and quantitative real-time PCR was performed using SsoFast Eva Green Supermix kit (Bio-Rad). β-actin was used as an internal normalization control.

### Immunoblotting

HCT-116 cells and primary colon cancer cells were cultured in McCoy’s 5A Medium with 1% FBS or in serum-free medium in the absence or presence of IL-1β for seven days. Cells were then washed with cold PBS, lysed in RIPA buffer [25 mM Tris–HCl (pH 7.6), 150 mM NaCl, 1% NP-40, 1% sodium deoxycholate, 0.1% SDS) and pelleted by centrifugation. Protein concentrations were determined using a NanoDrop instrument (Thermo Scientific, Wilmington, DE). Cell lysates (30 μg protein for each sample) were incubated for 5 min at 100^0^C in 2x loading buffer, separated by electrophoresis in 10% polyacrylamide gels, and transferred to PVDF membranes (Millipore, Bedford, MA). Membranes were blocked with 5% milk in TBST and then incubated with primary antibodies. The anti-Zeb1, anti-Nestin and anti-Bmi1 (H99) antibodies were from Santa Cruz biotechnology (Santa Cruz, CA). The anti-E-cadherin and anti-Bmi1 clone F6 antibodies were from Millipore and anti-β-actin antibody was from Sigma. After washing with TBST, the membrane was incubated with one of the two secondary antibodies, HRP-conjugated goat anti-mouse IgG-HRP (Millipore) or anti-rabbit IgG HRP-linked antibody (Cell Signaling, Danvers, MA). Detection was performed using HyGLO substrate (Denville Scientific, Metuchen, NJ) and images were taken using an AlphaEaseFC imaging system (Cell biosciences, Santa Clara, CA). The graph digitizing software UN-SCAN-IT (Silk Scientific, Orem, Utah) was used to quantify intensities of protein bands.

### Infection with shRNA lentiviral particles

HCT-116 cells were cultured in McCoy’s 5A Medium with 10% FBS until they became 50% confluent. Then cells were infected with scrambled or Zeb1 shRNA lentiviral particles (Santa Cruz) as described by the manufacturer. Stable infected cells were established via selection with puromycin (10 μg/ml).

### Statistical analysis

Student’s *t* test was used to determine statistical significance for all analyzed data. We consider a two-sided p < 0.05 as significant.

## Competing interests

The author(s) declare that they have no competing interests.

## Authors’ contributions

YL, LW, and JS designed the experiments. YL, LW, LP, and AGB performed experiments. LW, AGB, LP, and JS wrote the manuscript. All authors approved the final draft of this manuscript.
